# Integration of Multi-Sensor Fusion and Decision-Making Architecture for Autonomous Vehicles in Multi-Object Traffic Conditions

**DOI:** 10.3390/s25227083

**Published:** 2025-11-20

**Authors:** Hai Ngoc Nguyen, Thien Nguyen Luong, Tuan Pham Minh, Nguyen Mai Thi Hong, Kiet Tran Anh, Quan Bui Hong, Ngoc Pham Van Bach

**Affiliations:** 1 Vietnam National Space Center, Vietnam Academy of Science and Technology, Hanoi 100000, Vietnam; nnhai@vnsc.org.vn (H.N.N.); nlthien@vnsc.org.vn (T.N.L.); pmtuan@vnsc.vast.vn (T.P.M.); mthnguyen@vnsc.org.vn (N.M.T.H.); 2 Department of Mechanical Engineering, Johns Hopkins University, Baltimore, MD 21218, USA; trananhkiet1410@gmail.com; 3 Faculty of Information Technology, University of Engineering and Technology—VNU, Hanoi 100000, Vietnam; 22028016@vnu.edu.vn

**Keywords:** multi-sensor fusion, data fusion, autonomous vehicle, vision and sensors

## Abstract

Autonomous vehicles represent a transformative technology in modern transportation, promising enhanced safety, efficiency, and accessibility in mobility systems. This paper presents a comprehensive autonomous vehicle system designed specifically for Vietnam’s traffic conditions, featuring a multi-layered approach to perception, decision-making, and control. The system utilizes dual 2D LiDARs, camera vision, and GPS sensing to navigate complex urban environments. A key contribution is the development of a specialized segmentation model that accurately identifies Vietnam-specific traffic signs, lane markings, road features, and pedestrians. The system implements a hierarchical decision-making architecture, combining long-term planning based on GPS and map data with short-term reactive planning derived from a bird’s-eye view transformation of segmentation and LiDAR data. The control system modulates the speed and steering angle through a validated model that ensures stable vehicle operation across various traffic scenarios. Experimental results demonstrate the system’s effectiveness in real-world conditions, achieving a high accuracy rate in terms of segmentation and detection and an exact response in navigation tasks. The proposed system shows robust performance in Vietnam’s unique traffic environment, addressing challenges such as mixed traffic flow and country-specific road infrastructure.

## 1. Introduction

Autonomous vehicles (AVs) have emerged as a revolutionary advancement in transportation technology, promising to enhance road safety, optimize traffic flow, and transform urban mobility. While significant progress has been made in autonomous driving systems for well-structured environments in developed countries, the implementation of AVs in diverse traffic conditions, particularly in Southeast Asian countries like Vietnam, presents unique challenges. These challenges include heterogeneous traffic patterns, varying road infrastructure quality, complex traffic sign systems, and distinct driving behaviors [1]. Traditional autonomous driving approaches often struggle in Vietnam’s traffic environment due to several factors. First, the traffic flow is characterized by a mix of vehicles including cars, motorcycles, bicycles, and pedestrians, creating complex interaction scenarios. Second, lane markings and road infrastructure may be inconsistent or deteriorated, making traditional lane-following algorithms less reliable. Third, Vietnam’s traffic sign system includes unique elements that are not commonly addressed in existing autonomous driving datasets and models [2]. To address these challenges, this paper presents a comprehensive autonomous vehicle system specifically designed for Vietnam’s traffic conditions. Our system integrates multiple sensing modalities, including dual 2D LiDARs, camera vision, and GPS, to ensure robust environmental perception. The key innovations of our work include the following: a specialized segmentation model trained to recognize Vietnam-specific traffic elements, including unique traffic signs, lane markings, and road features; a hierarchical decision-making system that combines long-term route planning with reactive short-term navigation; a bird’s-eye view transformation approach that fuses segmentation results with LiDAR data for enhanced situational awareness; and a validated control system model that ensures stable vehicle operation across various traffic scenarios.

The results of our experiment, conducted on Vietnamese scenarios with different cases, demonstrate the system’s effectiveness in handling Vietnam’s unique traffic challenges. The proposed approach achieves significant improvements in comprehensive perception, navigation, and decision-making.

## 2. Related Works

### 2.1. Camera Segmentation and LiDAR Signal Representation

**Figure 1:**
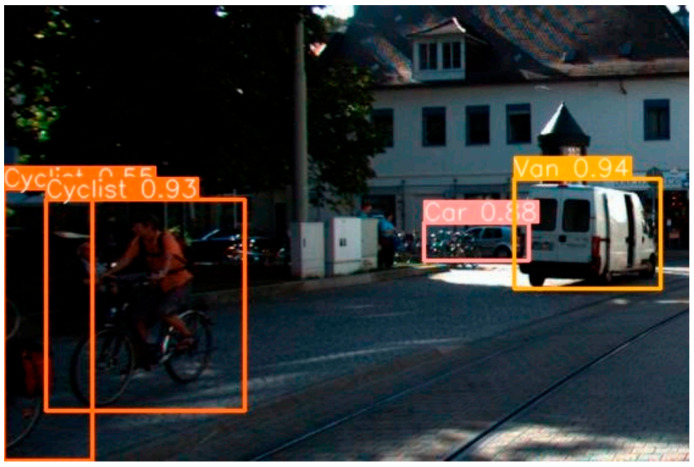
Vehicle and pedestrian detection [4].

Recent advances in autonomous vehicle perception have demonstrated the power of combining camera-based segmentation with LiDAR data. Camera segmentation using deep learning models, particularly YOLO, has shown exceptional performance in detecting and segmenting traffic elements like lanes, signs, vehicles, and pedestrians [3,4,5,6]. The evolution from previous YOLO versions, shown in Figure 1, has brought significant improvements in both accuracy and processing speed, making it suitable for real-time autonomous driving applications. However, autonomous vehicles need to have a comprehensive perception for several tasks, so it is necessary to apply a comprehensive model with multiple objects to improve input information for decision-making.

**Figure 2:**
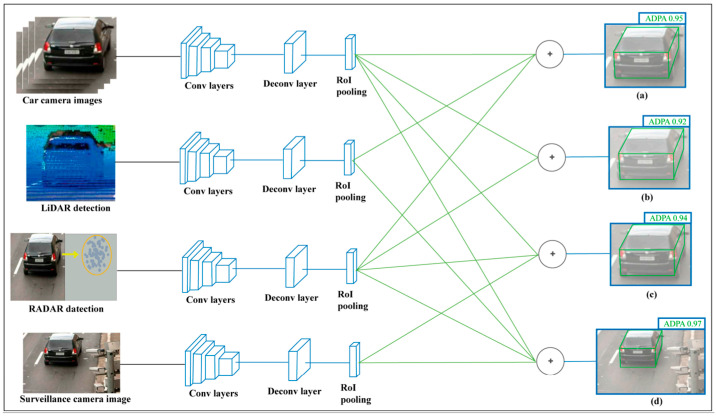
Sensor fusion with traffic surveillance camera system [7].

With LiDAR and camera fusion, Hasanujjaman et al. [7] proposed a sensor fusion approach that integrates AV onboard sensors with external traffic surveillance cameras to achieve 4D detection (height, width, length, and position), precise localization, and AI-based networking. Figure 2 shows their system, which uses convolutional neural networks for image processing and feature matching to enhance object detection and positioning. This fusion enables real-time data transmission and far monitoring, improving overall system reliability by leveraging surveillance cameras as anchor nodes for networking. While effective for external augmentation, this method relies on position-dependent infrastructure, which may limit its applicability in areas without dense camera coverage.

**Figure 3:**
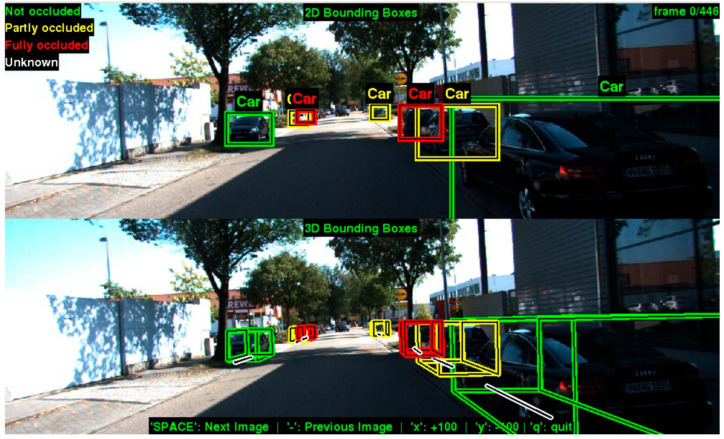
Camera and LiDAR 2D fusion for precise distance estimation [10].

Several studies have explored effective ways to represent and fuse LiDAR data with camera segmentation. In different research [8], researchers developed a method to combine geometric information from LiDAR with semantic segmentation from cameras to create a more comprehensive understanding of the environment. The fusion of these complementary sensor modalities helps overcome the limitations of each sensor type—cameras provide rich semantic information but lack precise depth measurements, while LiDAR provides accurate spatial information but lacks semantic context [9]. With this method, the authors have demonstrated a precise method for obstacle distance estimation, and this method can be improved by combining a comprehensive model and image processing. Figure 3 shows an example of lidar-vision fusion:

Bird’s-eye view (BEV) representation has emerged as a particularly effective approach for autonomous driving perception. Prakash et al. [11] demonstrated a multi-modal fusion transformer that effectively combines camera and LiDAR data into a unified BEV representation. Similar to our approach, Wang et al. [12] utilized multiple LiDAR sensors to create a comprehensive top-view model, though their work focused on highway scenarios rather than urban environments.

### 2.2. Decision-Making for Autonomous Vehicles

Decision-making in autonomous vehicles typically follows a hierarchical structure, separating long-term strategic decisions from short-term tactical controls [1,13]. Comprehensive surveys of decision-making architectures highlight the importance of integrating both rule-based and reactive approaches [14]. Research emphasizes that effective autonomous driving requires both adherence to traffic rules and responsive behavior to dynamic obstacles. For long-term decision-making, recent work has focused on incorporating map information and traffic rules into planning frameworks. Studies propose strategic planning systems that consider both static rules (from traffic signs and road markings) and dynamic conditions (from real-time perception) [15]. This approach aligns with our system’s use of GPS and detected signs for long-term planning. In the domain of short-term decision-making, several approaches have emerged for converting perception inputs into control commands. Recent studies have explored integrated frameworks that jointly design path planning and control to enhance real-time performance and robustness [16]. These approaches couple the trajectory generation process directly with the controller, improving smoothness, stability, and responsiveness in dynamic traffic scenarios. Other research has focused on robust cooperative strategies for multi-vehicle coordination in unsignalized intersections, where robust output-feedback control ensures safe interactions among connected vehicles despite communication and sensing uncertainties [17]. In addition, output-feedback path-tracking controllers have been proposed to maintain vehicle stability and steering accuracy even in the presence of actuator faults, providing fault-tolerant performance for distributed electric vehicles [18].

**Figure 4:**
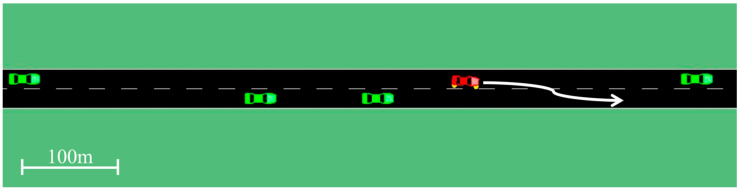
Decision-making by RL-based system supports [20].

Beyond control robustness, system-level vulnerability has been analyzed through vibration-theoretic models that quantify how disturbances propagate through nonlinear vehicle platoons, providing a theoretical foundation for resilience in connected-vehicle systems [19]. These studies collectively indicate that effective autonomous decision-making requires integration across perception, planning, and control layers to manage uncertainty and maintain safety. Lin et al. [20] showed that a Deep Reinforcement Learning (DRL) framework, using domain randomization of microscopic traffic models (IDM, MOBIL), improves sim-to-real transfer for AV control in complex freeway scenarios. This RL-based system supports adaptive decision-making for heterogeneous traffic, and the results are demonstrated in Figure 4. However, it primarily focuses on control in simulated settings with comprehensive assumptions, while the realistic system cannot provide perfect input for the model.

However, in Vietnam, traffic is characterized by mixed vehicle types, irregular lane markings, and frequent unexpected movements. The proposed hierarchical decision-making system addresses these challenges by integrating a long-term planner based on traffic rules and route data with a short-term reactive layer that dynamically assesses risk from fused LiDAR–camera perception. This design enhances responsiveness and safety in unstructured and high-density traffic conditions.

### 2.3. Route Planning and PathFinding

Route planning and local pathfinding are fundamental components of autonomous navigation. Traditional graph-based algorithms such as A* and Dijkstra remain widely used due to their reliability and efficiency in static road networks, while recent improvements incorporate real-time traffic data, detour costs, and safety constraints to enhance route optimization [21,22,23]. Reinforcement-learning-based planners have also been applied to dynamically adjust routes based on traffic flow, travel time, and changing environmental conditions [22]. Integrated planning–control frameworks have further advanced route optimization by embedding trajectory generation within the control structure, ensuring smooth transitions between planning and execution while reducing computational latency [16]. Radar-based perception has also become increasingly important for route safety under visual conditions. By fusing radar and vision data, recent systems achieve robust detection of pedestrians and vehicles, supporting safer local planning and collision avoidance [24].

At the cooperative level, robust output-feedback trajectory-tracking methods enable multiple connected vehicles to coordinate motion and maintain safe spacing at intersections without centralized control [17]. Sampling-based planners such as RRT and PRM remain popular for obstacle avoidance in continuous spaces, though their computational complexity limits applicability in embedded systems [25,26].

In Vietnam and other countries in Southest Asia, urban conditions involve dense mixed traffic, inconsistent lane structures, and spontaneous obstacles. To address these challenges, the proposed system integrates a lightweight A*-based global planner with a short-term reactive controller informed by real-time sensor fusion. This combination provides computational efficiency while maintaining adaptability and safety in complex and unstructured traffic environments.

### 2.4. Novelty of the Proposed Approach

While the reviewed works advance individual components of AV systems—such as camera segmentation with YOLO [3,4,5,6], domain-randomized RL for adaptive decision-making [20], feature-fused marking detection, and external camera fusion for networking [4,7]—they often operate in isolation or rely on assumptions not suited to diverse traffic challenges, including mixed vehicle-pedestrian flows, inconsistent lane markings, and variable infrastructure.

The proposed system introduces a comprehensive multi-sensor fusion model that segments a wide range of traffic objects (e.g., lanes, vehicles, pedestrians, signs) by fusing data from dual 2D LiDARs, camera vision, and GPS, with direct onboard LiDAR fusion independent of external positioning like surveillance cameras [7]. This ensures perception without infrastructure dependencies. For decision-making, we combine hierarchical long-term planning (leveraging GPS, map data, traffic sign detection, and lane segmentation for route conditions) with short-term reactive control (using bird’s-eye view fusion of segmentation and LiDAR data). The experimental results validate the system’s performance in real Vietnamese traffic, filling the need for end-to-end architectures.

## 3. System Architecture and Implementation

### 3.1. System Architecture Proposal

**Figure 5:**
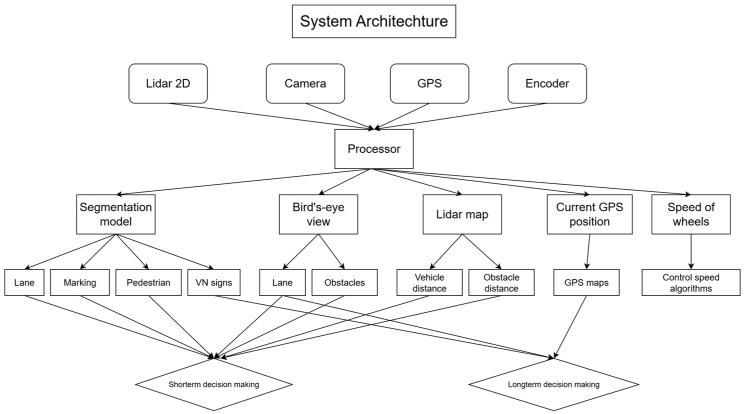
System architecture for the autonomous vehicle.

The autonomous vehicle system architecture integrates multiple sensor modalities, including 2D LiDAR, camera, GPS, and wheel encoders, to enable perception, localization, and decision-making. All the information from the sensors is processed for several tasks simultaneously. The proposed architecture for the system is shown in Figure 5 below:

For segmentation and detection, the returned results are applied for short-term decision-making with masks of important objects on the road. The detected Vietnamese traffic signs are analyzed, then those analyses are sent to the long-term decision-making model alongside GPS data and the segmentation model by ROS. The long-term decision-making model receives information from ROS nodes and sets the traffic restrictions (speed limits, turning prohibited, …) and the direction by GPS and lane segmentation.

The masks of objects on the road are fused with 2D LiDAR signals to improve the perception of the vehicle. The top view is dedicated to a visualization of the vehicle’s front view, which can be analyzed for short-term decision-making. GPS sensors are used for pathfinding and vehicle positioning. The encoder from each wheel provides information about the velocity, acceleration, and direction of the vehicle, which is essential for the control system. The short-term model uses the above exploitation to understand current states then returns the control decisions (speed, steering angle, …).

### 3.2. Implementations

#### 3.2.1. YOLOv8 Instance Segmentation and 2D LiDAR Fusion and Perception Visualization

**Figure 6:**
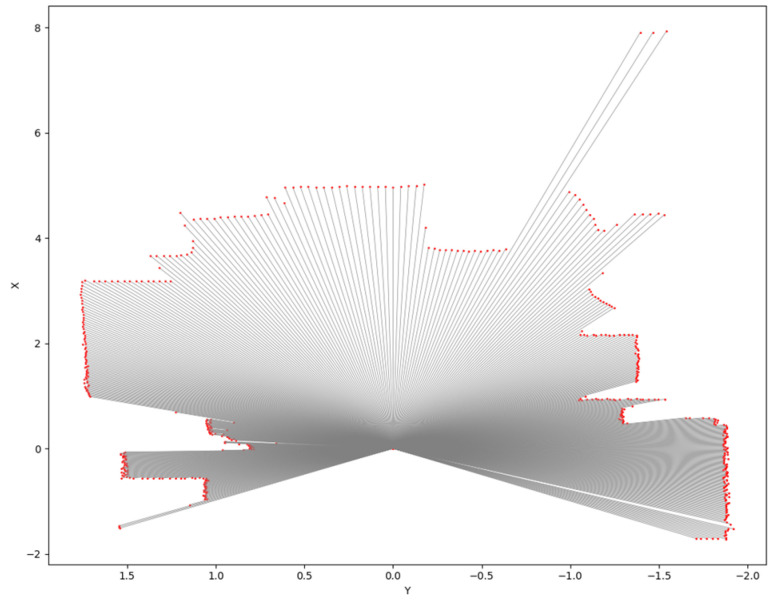
A mask from 2D LiDAR of the autonomous vehicle.

A 2D LiDAR sensor can be exploited to perceive the surrounding environment by analyzing the distance measurements from its emitted rays. The 2D LiDAR sensor provides 541 distance values, each corresponding to a ray spaced at 0.5-degree intervals and covers a total field of view of precisely 270 degrees. By converting these polar coordinates (angle and distance) into Cartesian coordinates (X, Y), a point cloud representation of the environment can be constructed. A converted frame of the LiDAR signal is expressed in Figure 6. This data can be processed to detect obstacles and understand the spatial layout of the surroundings. By continuously updating and analyzing the point cloud over time, the sensor can assist in real-time decision-making for navigation and path planning.

**Figure 7:**
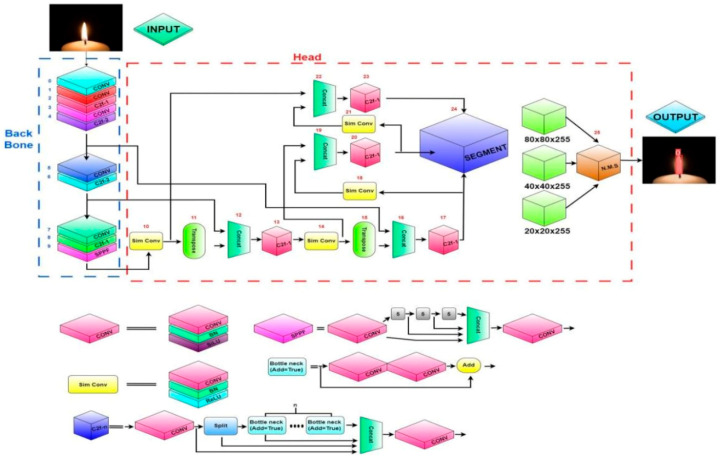
YOLOv8 segmentation model [27].

For comprehensive segmentation and detection, we used YOLOv8 segmentation for lanes, markings, vehicles, and pedestrians. The complexity of the multiple feature extraction layers of the YOLOv8 model is illustrated in Figure 7, ensuring its applicability to the segmentation for autonomous vehicles. Lane segmentation is applied for short-term decision-making by determining the accepted area to move. Marking segmentation is dedicated to analyzing the acceptance of lane changing. Vehicle and pedestrian segmentations collaborate with 2D LiDAR analysis to determine accurate distances to the autonomous vehicle.

For traffic sign detection, we collect and label Vietnamese traffic signs, then train with YOLOv8-s detection model. The dataset of Vietnamese traffic signs is collected with respect to Vietnamese traffic rules. The collected ones are important for navigation and speed of the autonomous vehicle, which requires consistency between vehicles on the specific road.

**Figure 8:**
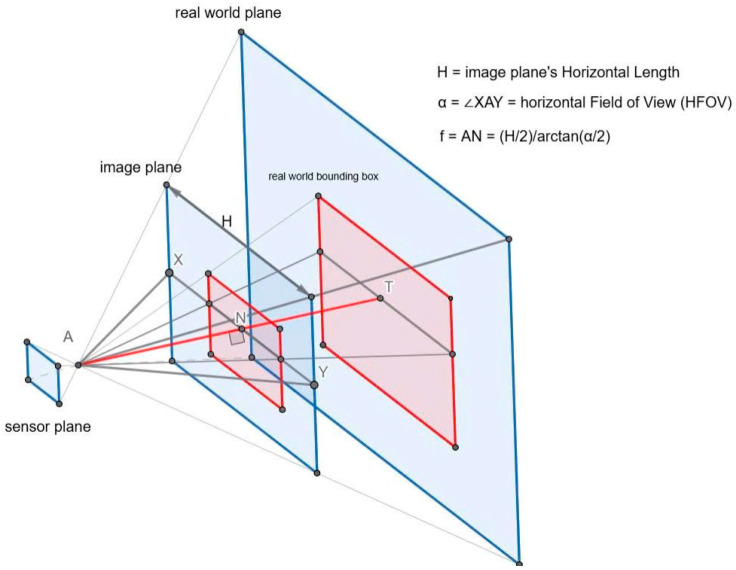
Camera view for object’s angle estimation.

The signal of the LiDAR, segmentation masks, and the top-view model are fused to create a comprehensive perception for the autonomous vehicle. First, vehicles and pedestrians are considered obstacles to the autonomous vehicle. From segmentation masks of vehicles and pedestrians, we determine the angular range of the object relative to the camera. In [28], the author calculates that the angle of incidence of an object is a linear function of the pixel coordinates. However, this calculation is only valid when the camera sensors are arranged in a spherical shape or in a rectangular layout with a narrow field of view. The relationship between the sensor plane and the real-world plane is illustrated in Figure 8 as discussed in [29]. We compute it as follows:

Consider a camera with a horizontal field of view (HFOV) of α degrees and a horizontal resolution of H pixels. The half-HFOV is α/2, and the image width is H, meaning half of the image corresponds to H/2 pixels. The focal length f in pixels can be derived as [29]:

(1) $$f=\frac{H}{2\tan\left(\frac{\alpha }{2}\right)}$$

The principal point, corresponding to the 0-degree angle, is located at the center of the image [29]:

(2) $$c_{x}=\frac{H}{2}$$

where $c_{x}$ denotes the center coordinate of the image.

$x_{1}$ and $x_{2}$ are the left and right pixel coordinates of the detected object’s bounding box. By convention, the extreme right of the image corresponds to an angle of $\frac{\alpha }{2}$, while the extreme left corresponds to $\frac{-\alpha }{2}$. The angles subtended by the object’s left and right boundaries are given by [29]:

(3) $$\theta_{1}=\tan^{-1}\left(\frac{x_{1}-C_{x}}{f}\right)$$

(4) $$\theta_{2}=\tan^{-1}\left(\frac{x_{2}-C_{x}}{f}\right)$$

After determining the potential angles of obstacles, the potential angles are converted to two potential rays from LiDAR by:

(5) $$ray_{potential\_left}=2x(\theta_{1}+135)$$

if the determined rays are on the right compared to the center of the camera.

(6) $$ray_{potential\_left}=2x(135-\theta_{1})$$

if the determined rays are on the left compared to the center of the camera.

(7) $$ray_{potential\_right}=2x(\theta_{2}+135)$$

if the determined rays are on the right compared to the center of the camera.

(8) $$ray_{potential\_right}=2x(135-\theta_{2})$$

if the determined rays are on the left compared to the center of the.

Determined rays cannot be precise because of the limits of the camera and algorithm. However, the potential angle and rays of the obstacles are estimated; these can be applied to extract the precise rays for obstacles by:

(9) $$ray_{left}=dr_{i}\;\mathrm{if}\;\frac{dr_{i+1}-dr_{i}}{\theta_{i+1}-\theta_{i}}>Th$$

where: $ray_{left}$ is the precise ray on the left side of an obstacle. $dr_{i}s$ is the distance of ray number i. $\theta_{i}$ is the specific angle of ray number i.

After applying this formula for all potential rays on the left and right sides of the obstacles, we retrieve a set of values for differences between rays, and the two peaks on the left and right sides are the rays that accurately represent obstacles.

#### 3.2.2. Long-Short-Term Decision-Making Architecture Based on Sensor Exploitation

**Figure 9:**
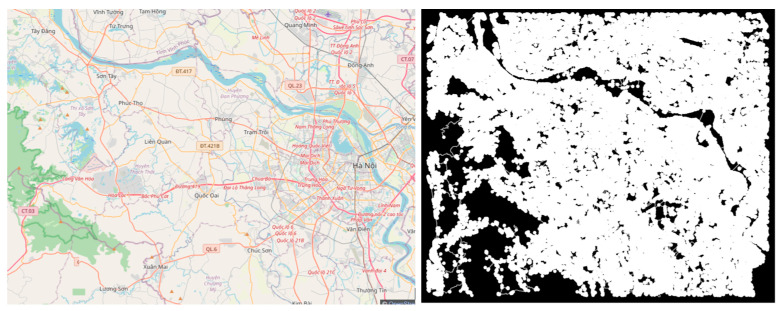
Hanoi city map and road layer (hidden).

Given the resource and data limitations of implementing route planning on an embedded OpenStreetMap shown in Figure 9, we opt for the traditional A* algorithm as the most practical and efficient choice for our application. To search for the path, we first input the geocode of the current position of the car and the name or geocode of the target point. The name of the target point is translated to geocode if available. These geocodes will be connected to the road map vertex. Next, we apply the A* algorithm to find the shortest path from the closest vertex of the start point to the closest vertex of the end point. 

**Figure 10:**
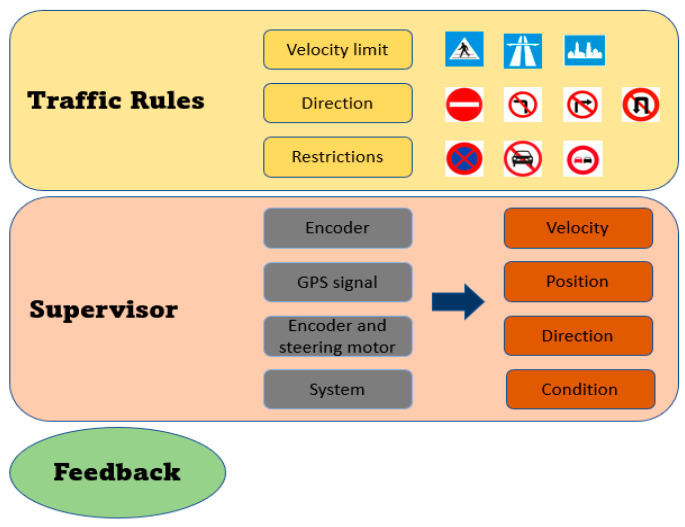
Schema for long-term decision-making of the autonomous vehicle.

Figure 10 illustrates the schema for long-term decision-making, which includes traffic rules, supervisor and feedback. Based on detected traffic signs, the vehicle automatically sets the restrictions and conditions based on Vietnamese traffic rules. The supervisor collects information about velocity, position, direction, and vehicle conditions to ensure the system respects the rules and sends feedback to the control system. The traffic rules are set based on the detected Vietnamese signs. The restrictions and conditions conveyed by the traffic signs are processed by traffic rules. These rules ensure that vehicles progress consistently without traffic violations. The supervisor plays an important role in short-term decision-making, processing the conditions and restrictions to ensure they are applied properly in the autonomous system. Afterward, the checking results and system requirements are sent to the short-term decision-making model for execution. The long-term decision-making model is designed specifically for Vietnamese traffic conditions, where the meaning of signs should be combined for precise decision-making. For turning prohibitions, their validity ends after intersections or turning branches on the road. Meanwhile, the restrictions and conditions from areas and one-way signs remain active.

**Figure 11:**
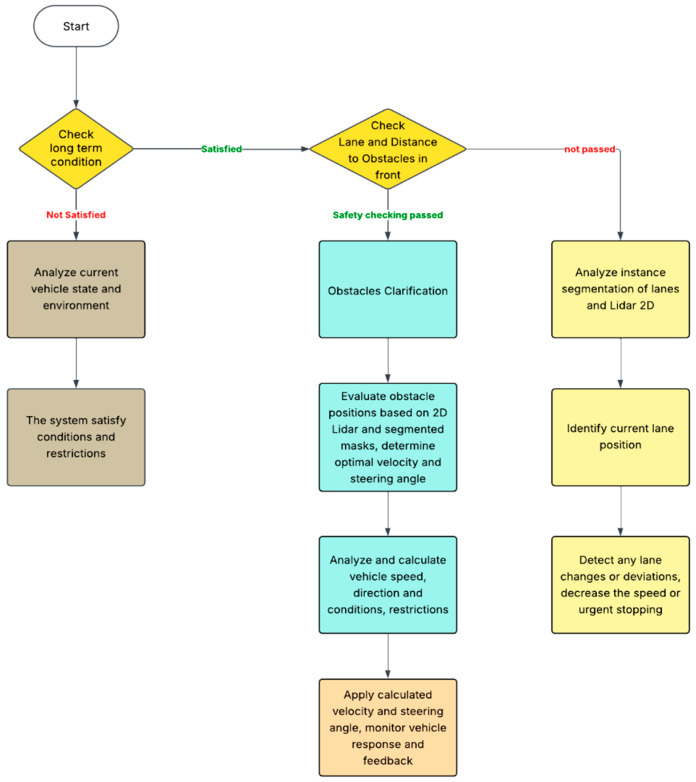
Flowchart for short-term decision-making of the autonomous vehicle.

In the short-term decision-making process described in Figure 11, the vehicle continually evaluates its immediate surroundings and adjusts its trajectory and speed to ensure safe and efficient navigation. After verifying that long-term conditions and restrictions are satisfied, the system proceeds to assess the lane geometry and detect obstacles using sensors such as LiDAR and cameras. It then determines the optimal velocity and steering angle by analyzing real-time data on lane positions, distances to nearby vehicles or objects, and any potential lane deviations or urgent stopping scenarios [30,31]. If the analysis indicates unsafe conditions, the system refines its perception through instance segmentation and updates its understanding of the environment accordingly. The capability of vehicles to adapt to Vietnamese traffic conditions, where obstacles occasionally are not perceived by the model or appear unexpectedly from alleys, is improved. The signal from LiDAR is not only used in combination with the camera to determine precise distances to objects, but it also ensures the safe distance between the vehicle and the surrounding environment. Although in our system LiDARs always return distances at an angle of 270 degrees, the risks assessed depend on the vehicle’s moving direction, which means a larger distance in the moving direction can still increase the risk more than a smaller distance to the sides or rear of the vehicle. Therefore, we declare β a risky coefficient with respect to different $n_{rays}$ calculated by:

(10) $$\beta_{i}=\frac{1}{\sigma \sqrt{2\pi }}\exp\left(-\frac{{\left(\theta_{i}-\mu \right)}^{2}}{2\sigma^{2}}\right)$$

where $\beta_{i}$ is the risky coefficient with respect to index i. σ is the standard deviation (in 60 degrees), controlling the spread of emphasis. $\theta_{i}$ is the angle of the ray index i. μ is the mean angle shift (in degrees), representing the turning direction.

We consider all the unfiltered rays from LiDARs to belong to the surrounding environment, where the distances are calculated in a cluster to evaluate the surprising risks. The distance of individual rays is sensitive to tiny objects, so we use a sliding array to calculate the risk from unidentified objects to the vehicle.

(11) $$R=\frac{\sum_{n_{rays}}d}{n_{rays}\times \beta }$$

where R is the risk to the vehicle. d is the distance from an individual ray to LiDAR. $n_{rays}$ is the number of values in a sliding array. β is the risky coefficient with respect to different $n_{rays}$, equal to the average $\beta_{i}$ of n rays.

We calculate the risk using different sliding arrays and collect various risk values for the vehicle. Based on those risk estimations, the autonomous vehicle could make short-term decisions to ensure safety in unexpected traffic situations in Vietnam. Finally, the calculated control commands’ steering angle and velocity are applied, and feedback from the vehicle’s response is continually monitored to maintain safe driving performance. This loop of perception, analysis, and actuation occurs in rapid cycles to adapt to changing traffic conditions and complement the broader constraints set by the long-term decision-making framework. After short-term decision-making, the control system executes based on processed information. To achieve efficient turning, we follow Ackermann steering geometry, which ensures that all wheels follow circular paths around a common instantaneous center of rotation (ICR). To further optimize efficiency, we adjust each wheel’s velocity to match the expected velocity profile dictated by Ackermann steering.

Our inputs are turning angle $\left(\delta \right)$ and current speed. From the turning angle, we compute the central turning radius that the center of mass should follow:

(12) $$R=\sqrt{a^{2}+l^{2}\cot^{2}(\delta )}$$

where a is the lateral offset (if applicable). l is the wheelbase (distance between front and rear axles). cot(δ) is the cotangent of the steering angle.

To compute the turning radius for each wheel, we determine R, then compute the radii for each wheel as follows:

Rear Axle Center Radius:

(13) $$R_{rear,center}=\sqrt{R^{2}-\frac{l^{2}}{4}}$$

Rear Wheel Radii:

(14) $$R_{rear,inner}=R_{rear,center}-\frac{\omega }{2}$$

(15) $$R_{rear,outer}=R_{rear,center}+\frac{\omega }{2}$$

Front Wheel Radii:

(16) $$R_{front,inner}=\sqrt{R_{rear,inner}^{2}+l^{2}}$$

(17) $$R_{front,outer}=\sqrt{R_{rear,outer}^{2}+l^{2}}$$

where ω is the track width of the vehicle.

The kinematic design of vehicle steering systems impacts handling, stability, and tire wear. Three common configurations are parallel steering, Ackermann steering, and anti-Ackermann steering, each suited to different applications. Parallel steering turns both front wheels at the same angle. While simple to implement, it causes tire scrubbing and excessive wear, making it impractical for most vehicles. It finds limited use in autonomous robotic platforms where wheel slip is negligible. Ackermann steering ensures that the inner and outer wheels follow concentric paths during a turn, reducing slip and improving traction. It enhances maneuverability and reduces tire wear, making it ideal for road vehicles. However, it can contribute to understeer at high speeds and requires precise linkage design.

Anti-Ackermann steering, where the outer wheel turns more than the inner wheel, is used in high-speed racing to optimize tire load distribution and improve cornering grip. While beneficial for performance, it increases low-speed tire scrubbing and is unsuitable for regular road vehicles. Parallel steering is simple but inefficient. Ackermann steering is best for general vehicles, balancing maneuverability and tire wear. Anti-Ackermann steering benefits high-speed racing but is impractical for normal driving due to increased tire wear.

## 4. Experiments and Results

### 4.1. Results of YOLOv8 Instance Segmentation and 2D LiDAR Fusion and Top View for Vehicle Front-View Visualization

**Figure 12:**
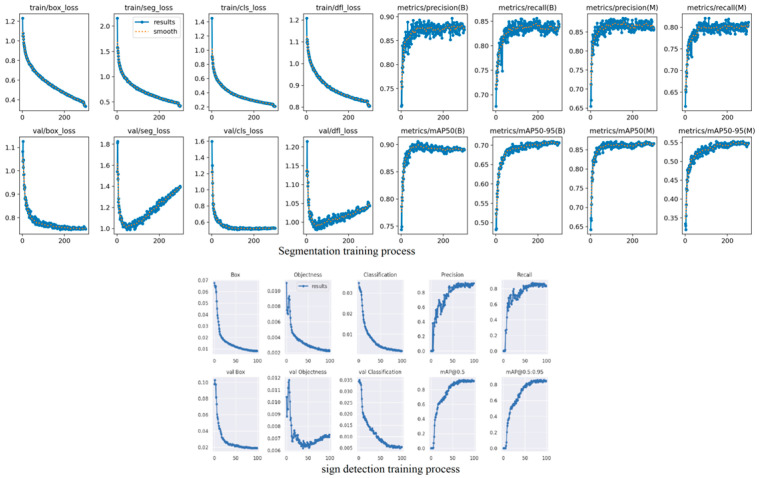
Graphs of segmentation and detection training process.

We developed and trained two deep learning models based on datasets independently collected and annotated to reflect the unique characteristics of the Vietnamese traffic environment. The first dataset focuses on instance segmentation and includes four key classes: lane markings, road markings, pedestrians, and vehicles (containing different kinds of vehicles in the Vietnamese traffic environment)—capturing the complexity of real-world road scenarios in Vietnam. The second dataset is dedicated to Vietnamese traffic signs, covering diverse and localized sign types under varying lighting and environmental conditions. As a result, we conducted two separate training processes—one for segmentation and one for detection—whose performance is visualized in the respective graphs. The graphs in Figure 12 indicate a good training process; the accuracy of the detection model is approximately 97% for traffic signs, and the segmentation model acquired favorable metrics: precision: 92%, accuracy: 95%, and mAP90: 0.75.

**Figure 13:**
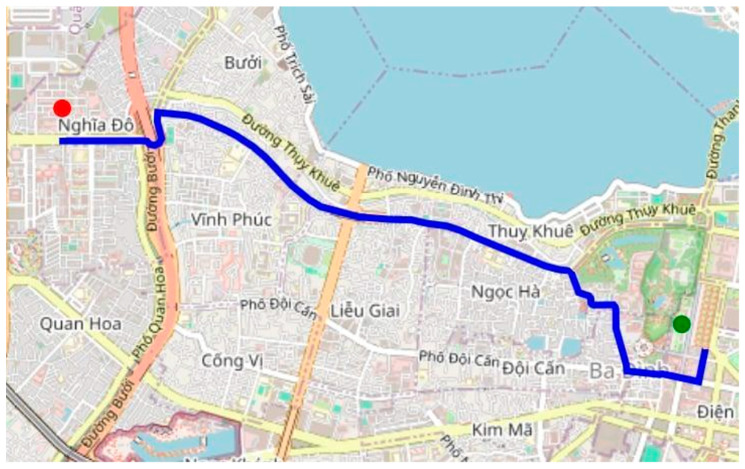
Pathfinding on the map in Hanoi.

The A* algorithm successfully searches for a route from the Vietnam Academy of Science and Technology to the President Ho Chi Minh Mausoleum, as shown in Figure 13. The generated path was efficient in terms of distance and computational time, demonstrating the algorithm’s effectiveness in urban route planning.

**Figure 14:**
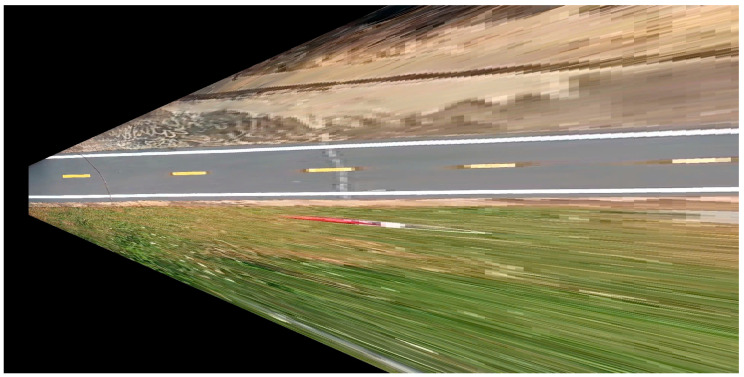
Result for top-view of the autonomous vehicle.

Modeling top-view plays an important role in controlling the system, where the pathfinding algorithms can be applied. In our results in Figure 14, we focus mainly on properties of the road and the objects on the road because they are segmented in the segmentation model. The front lane is estimated and combined with segmentation masks to find the optimal path for the vehicle.

**Figure 15:**
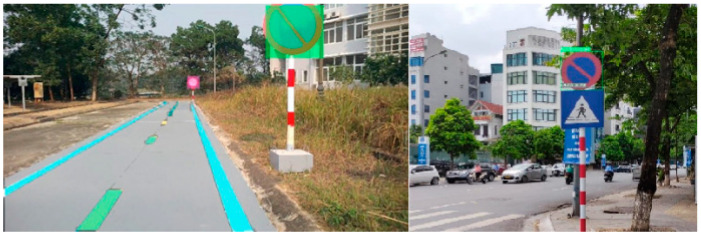
Result of traffic sign detection.

In the experimental scenarios shown in Figure 15, traffic signs are detected, and their meanings are subsequently analyzed by the long-term decision-making model. Based on this analysis, restrictions and regulations are applied to the short-term decision-making model. The signs are arranged as the Vietnamese traffic environment, where the system must integrate diverse traffic rule information due to the variety of vehicles on the road. The signs in the experimental scenarios are detected, and their meanings are consequently analyzed by the long-term decision-making model. The restrictions are set to the short-term decision-making model.

**Figure 16:**
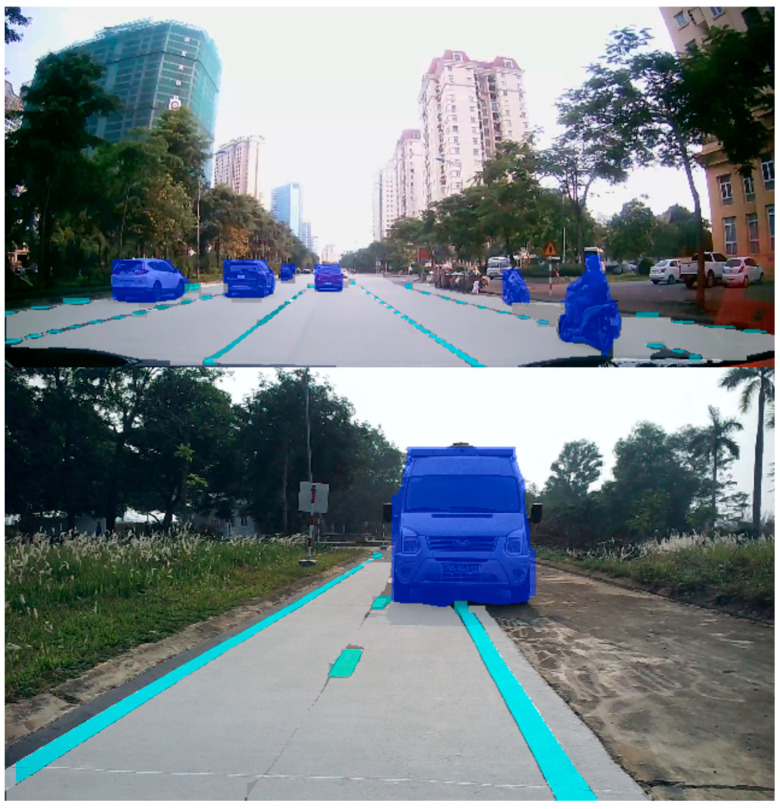
Result of lane, marking, and vehicle segmentation.

The segmentation model results are illustrated in Figure 16, where the masks precisely cover the instances in the image where the vehicle is in the middle of the road. Hence, those masks of instances can accurately be applied to different purposes. Our dataset was acquired in Vietnam’s traffic environment, where different lanes and vehicles can appear in a frame and the diverse segmentation of vehicles is required.

**Figure 17:**
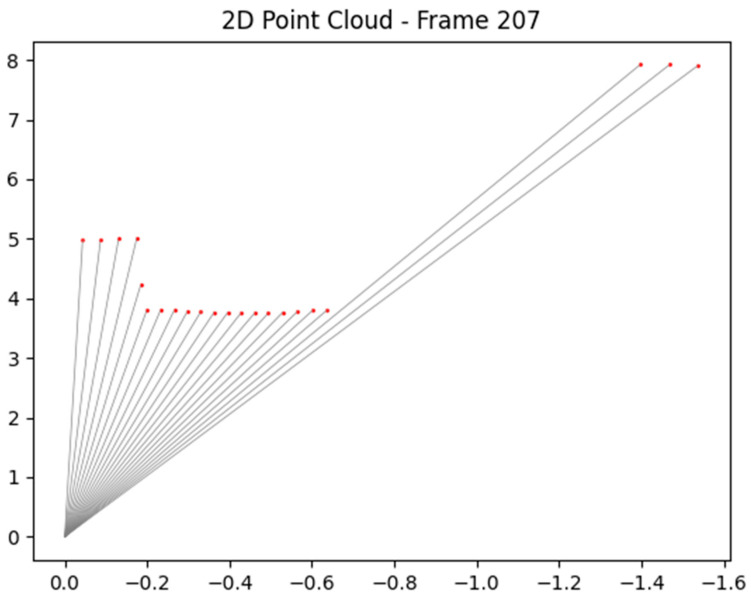
Selected rays for obstacle distance estimation from segmentation masks.

The potential angle of the detected object is exploited to extract precise rays regarding the object. Figure 17 shows the potential rays, whose values are applied to Formula (9) for extracting the precise rays of objects.

**Figure 18:**
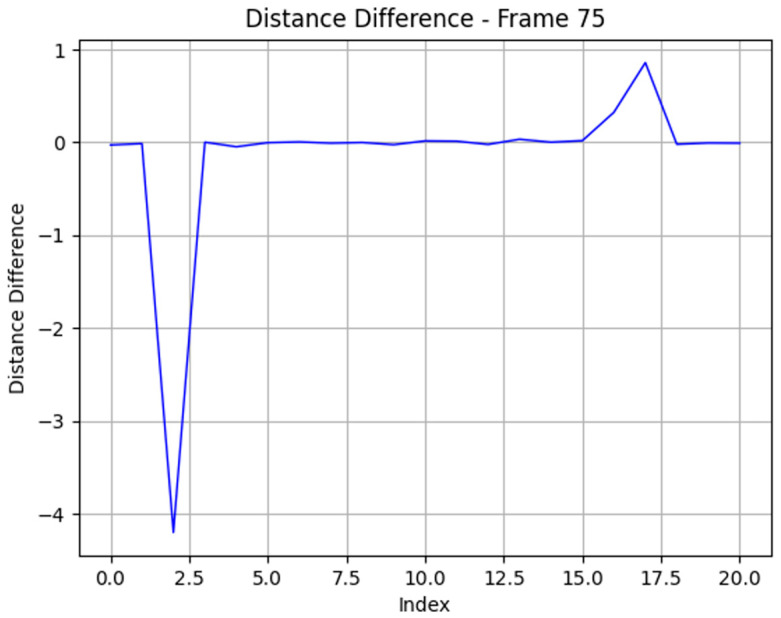
Two-dimensional LiDAR signal analysis from filtered obstacle rays from segmentation masks.

The precise rays reflecting the distance to objects are extracted by the difference between the distances of adjacent rays. Figure 18 indicates the role of filtering potential rays from the environment, where the peaks created by the objects’ rays are easily separated from others.

**Figure 19:**
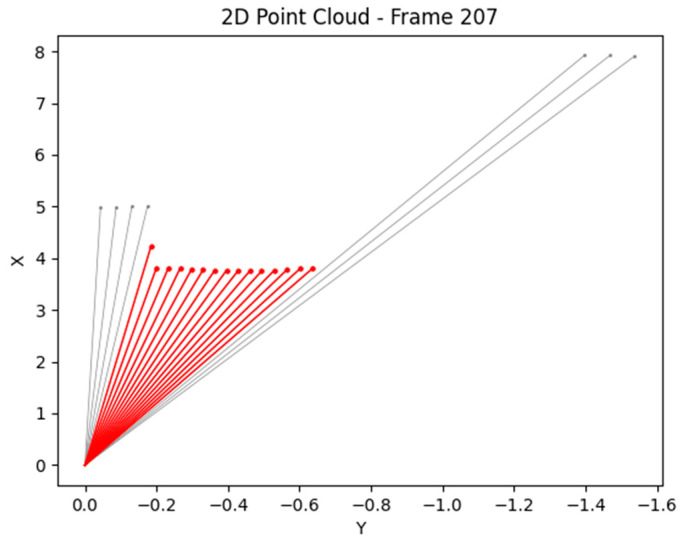
Precise rays represent the object.

In Figure 19, the extracted rays are colored red and precisely reflect the distance from Lidar 2D to the object. With precise distances, the vehicle’s system can evaluate the appropriate speed and steering angle.

### 4.2. Result of System Response

**Figure 20:**
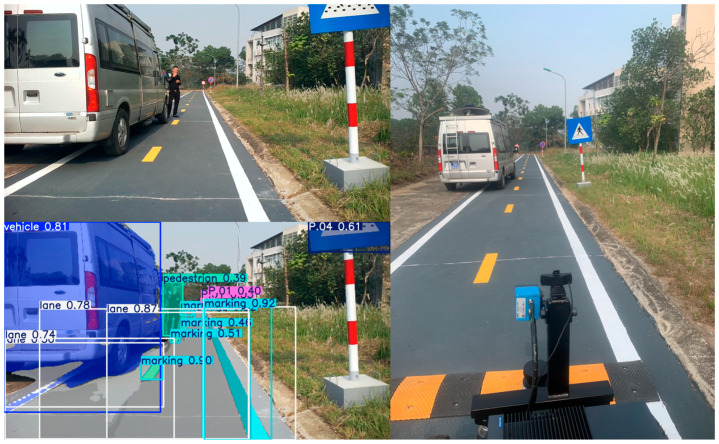
The system perceives objects and makes decisions.

**Figure 21:**
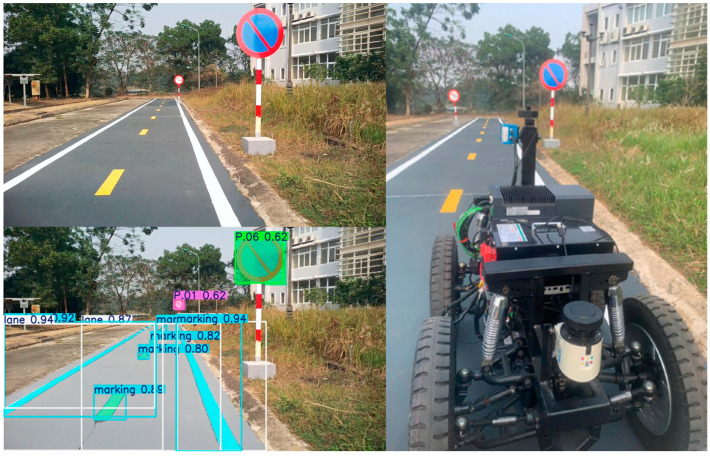
The system avoids objects and remains stable.

The system adapts well to multiple detected objects in the environment that the fusion model above perceives and analyzes. By evaluating the appropriate speed and steering angle, the vehicle can avoid detected obstacles, move to the proper lane, and evaluate the surrounding environment in real time. The results of experiments are shown in Figure 20 and Figure 21 below:

After overcoming obstacles and moving to the proper lane, the vehicle remains at a stable speed, respects the restrictions from the signs, and continuously collects and processes information.

**Figure 22:**
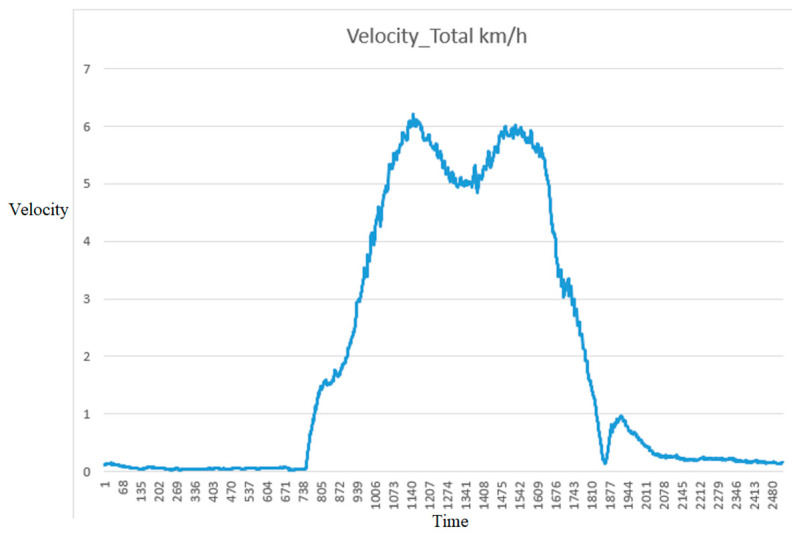
The vehicle’s velocity in an experiment.

In Figure 22, the graph shows a clear acceleration phase starting from rest, reaching a peak velocity of approximately 6 km/h. The car maintains high-speed operation with moderate fluctuations before decelerating smoothly to a near stop. Minor variations in velocity during the steady-state phase suggest adaptive control behavior, possibly in response to environmental factors or trajectory adjustments.

To meet the requirements of autonomous vehicles, we conducted experimentation with fine-tuned segmentation models. After a comprehensive comparison of model performance and accuracy, we selected YOLOv8-seg-m as our preferred model. The table below illustrates the hardware performance across different segmentation models, including our chosen YOLOv8-seg-m.

**Figure 23:**
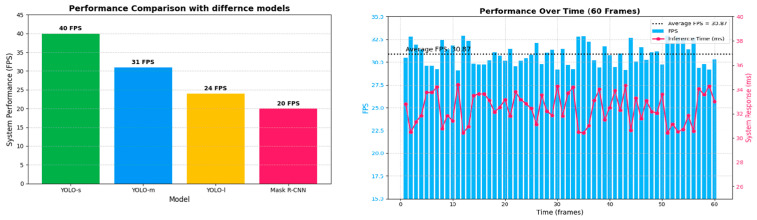
Hardware performance with different models and the selected model’s performance over time.

The performance evaluation with the YOLOv8-seg-m model is depicted in Figure 23. With the appropriate model, the system operated stably at approximately 31 FPS, which is sufficient for autonomous vehicles [32]. The inference time remains approximately 32 ms, ensuring timely responses of the autonomous vehicle across various scenarios.

## 5. Conclusions

This research presents the development and integration of a comprehensive autonomous vehicle system tailored to multi-object traffic conditions. By implementing multi-sensor fusion and a hierarchical decision-making architecture, the system demonstrates comprehensive multi-object perception, navigation, and control capabilities. The proposed models accurately segment lanes, markings, vehicles, and pedestrians, and detect Vietnam-specific traffic signs with 97% accuracy and a segmentation mAP of 0.75. After fine-tuning and model selection, the system operates stably at 31 FPS, ensuring robust situational awareness. The decision-making modules, integrating long-term route planning with short-term reactive behavior, enable the vehicle to navigate dynamically while adhering to traffic rules. Experimental results validate the system’s ability to perceive, plan, and act reliably in real-world environments, achieving stable vehicle operation, accurate obstacle avoidance, and compliance with traffic regulations. The outcomes demonstrate the system’s adaptability for deployment in diverse traffic scenarios in Vietnam.

The proposed model, which was comprehensively implemented in experiments, offers strong extensibility for broader AV applications and developments. The onboard multi-sensor fusion and segmentation pipeline can be fine-tuned with transfer learning on region-specific datasets to adapt to other traffic patterns or urban environments. Its hierarchical decision-making framework supports integration with collaborative perception in smart cities. Applicability extends to critical AV challenges, including adverse-weather robustness and regulatory compliance in mixed human–AV settings, accelerating safer autonomous mobility.

Although the proposed method was tested in several scenarios, its limitations include the lack of experiments under extreme weather conditions and the use of the custom dataset specific to Vietnamese traffic. Future work will focus on enhancing system precision in more scenarios with adverse weather conditions and improving stability.

## Data Availability

A part of project’s data can be found at: https://github.com/NgocHaiNguyen14/Auto_driving_car, accessed on 15 December 2024.

## Abbreviations

The following abbreviations are used in this manuscript:

LiDAR	Light Detection and Ranging
GPS	Global Positioning System
YOLO	You Only Look Once
BEV	Bird’s-eye view
ROS	Robot Operating System
